# Management of Scaphoid Nonunion With Iliac Crest Graft and K-wire Fixation: An Economically Viable Option

**DOI:** 10.7759/cureus.68826

**Published:** 2024-09-06

**Authors:** Tapan Kumar Das, Jitendra Mishra, Subham Panigrahy, Sunit Pani, Spandan Mishra

**Affiliations:** 1 Department of Orthopaedics, Institute of Medical Sciences and SUM Hospital, Bhubaneswar, IND

**Keywords:** bone grafting, displaced scaphoid nonunion, functional outcomes, humpback deformity, k-wire

## Abstract

Purpose

This study details the functional results, patient satisfaction, and cost-effectiveness of patients treated with Fisk-Fernandez surgery using iliac crest graft and K-wire for scaphoid nonunion.

Materials and methods

This study involved a retrospective analysis conducted between November 2022 and August 2024. Forty-two patients diagnosed with scaphoid nonunion were treated using a surgical approach that included autologous bone grafting combined with K-wire fixation to promote bone healing and stability. To enable comparison, the QuickDASH-9 score, visual analog scale (VAS), and patient-rated wrist evaluation (PRWE) score were used for both preoperative and postoperative evaluations at the final follow-up.

Results

Our study group received treatment for an average of 16 months post-injury, ranging from 6 to 28 months. The average time of union was six months, ranging from four to 18 months. The study significantly improved QuickDASH-9 scores, grip strength, PRWE scores, and VAS for pain. The study reported no complications, and all patients returned to their basic activities of daily living.

Conclusion

Results of this study show that displaced scaphoid nonunions can be successfully treated with K-wire fixation combined with iliac crest bone grafting utilizing the Fisk-Fernandez approach.

## Introduction

Scaphoid fractures comprise 50-80% of all carpal bone fractures in young individuals. Five to ten percent of nondisplaced scaphoid fractures result in nonunion [1]. A fall onto an outstretched hand in which the maximum impact occurs on the wrist is the typical cause of scaphoid fractures. Because these fractures might not be immediately visible on X-rays, diagnosing them can be difficult. The most common symptoms are wrist pain and swelling, particularly in the region just below the thumb and at the anatomical snuffbox. A timely and precise diagnosis is crucial in order to avoid problems that might cause chronic discomfort and reduced wrist function and dreaded complications such as humpback (excessive flexion) deformity, carpal collapse, nonunion, or avascular necrosis (AVN).

The scaphoid lacks periosteum, so healing occurs solely by primary intention. Scaphoid fracture fragments can also be displaced in multiple planes, further hindering healing [2]. Failure to unite at three months is known as scaphoid delayed union, while failure to unite at six months following scaphoid fracture is known as scaphoid nonunion. Ten percent or so of scaphoid fractures do not heal. The most important sign of scaphoid nonunion is that the patient experiences tenderness at the anatomical snuff box [3]. Correcting malalignment, offering a bridging wedge bone transplant, obtaining solid fixation, and applying mechanical compression are essential treatment tenets for scaphoid nonunion [4].

A palmar opening wedge graft is necessary in the majority of cases of nonunion with dorsal intercalated segmental instability of the carpus, as this causes a palmar flexion deformity of the scaphoid. Our research attempts to examine the functional results of the Fisk-Fernandez procedure in individuals with discomfort, instability, and reduced grip strength who have scaphoid nonunion [5-7].

## Materials and methods

A consecutive series of 46 patients with nonunion of scaphoid, admitted to the Department of Orthopaedics between November 2022 and August 2024 and operated on during this period, were retrospectively enrolled in the study. The study included all patients diagnosed with scaphoid nonunion (>6 months) who were skeletally mature and below 50 without wrist arthritis. The following patients were excluded from the study: patients with AVN changes in the scaphoid, associated fractures in the same limb, and those who did not consent to be included.

**Figure 1:**
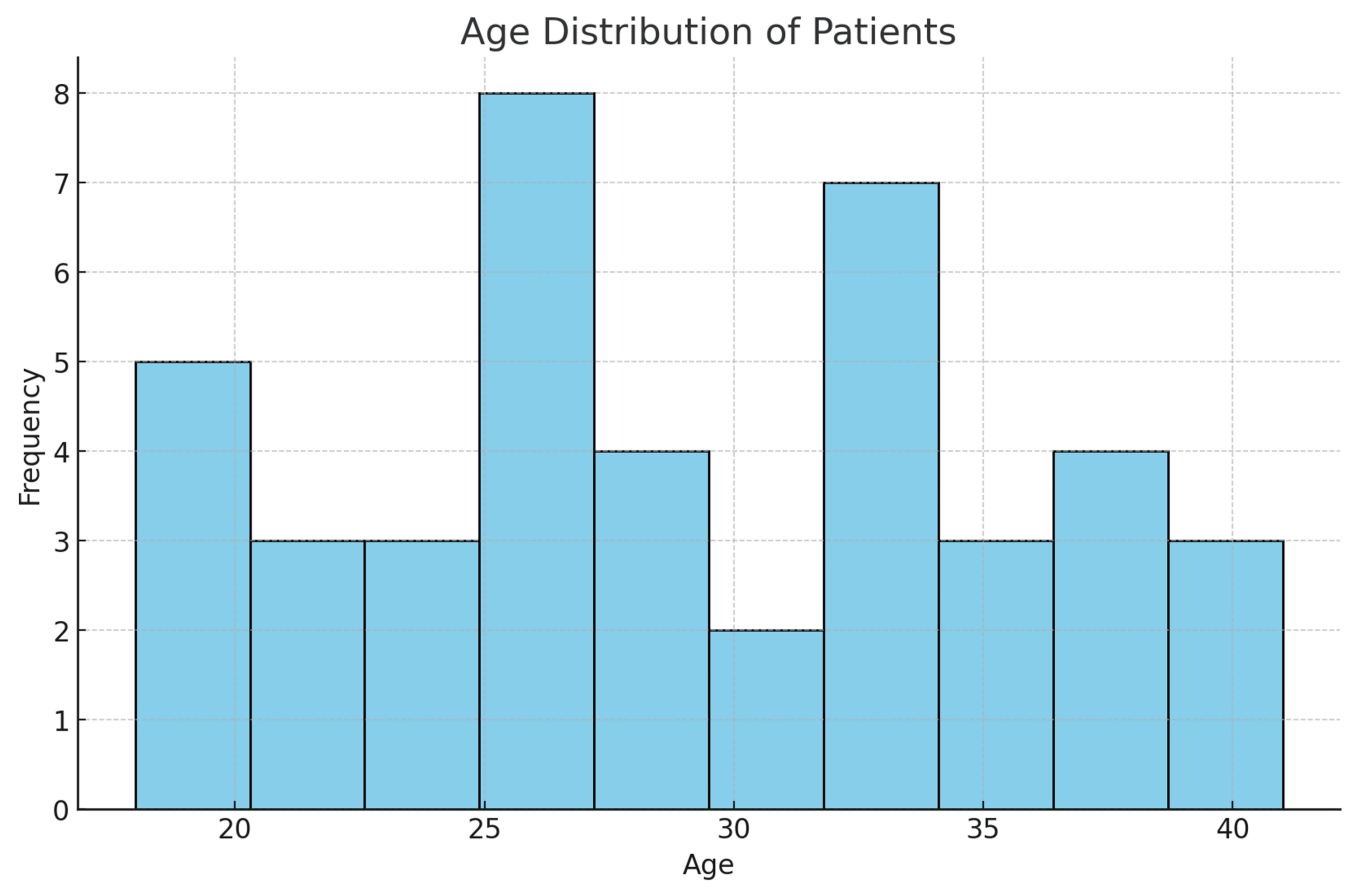
Age-specific distribution of patients presented with scaphoid nonunion

At last, 42 patients were added to the study: 20 men (47.61%) and 22 women (52.38%). Age-specific distribution is plotted in a histogram (Figure 1). All study participants gave their informed consent once the Institutional Ethics Committee of the Institute of Medical Sciences and SUM Hospital approved the study (approval number: IEC/IMS.SH/SOA/2022/820A). A single, experienced surgeon carried out each procedure. The patients were 29.05 years old on average. The study included retrospectively all patients who received the Fisk-Fernandez operation with K-wires between November 2022 and August 2024.

All patients with scaphoid nonunion fractures underwent intervention with a non-vascularized trapezoidal anterior wedge graft and were monitored over six months. X-ray radiographs of anteroposterior and lateral views were taken preoperatively and postoperatively. A CT scan was done to determine the fracture pattern, fracture gap, and degree of callus formation. An MRI was done to evaluate soft tissue status and identify AVN patients.

While the quantitative data was analyzed using averages and standard deviations, percentages and frequencies were used to represent categorical variables. The means obtained were compared using a paired t-test, where a p-value of less than or equal to 0.05 was deemed statistically significant.

Surgical procedure

The patients were planned for surgery after relevant investigations. Thirty surgeries were performed under brachial block anesthesia, with the remaining 12 being given general anesthesia using a sterile tourniquet on the mid-arm. A volar (palmar) approach along the flexor carpi radialis sheath followed by a wrist capsule was released for all cases.

**Figure 2:**
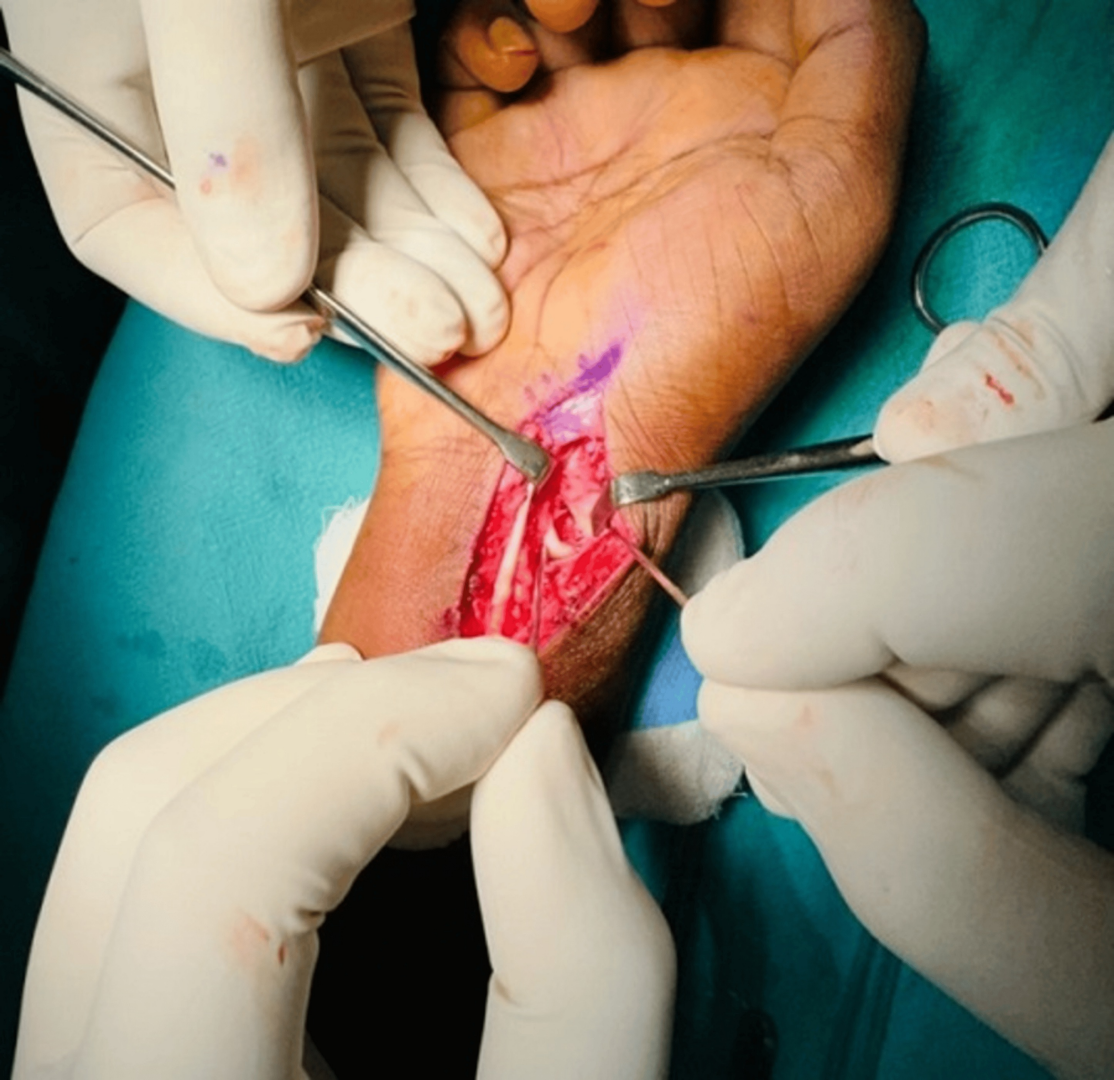
K-wires used to distract the nonunion site

**Figure 3:**
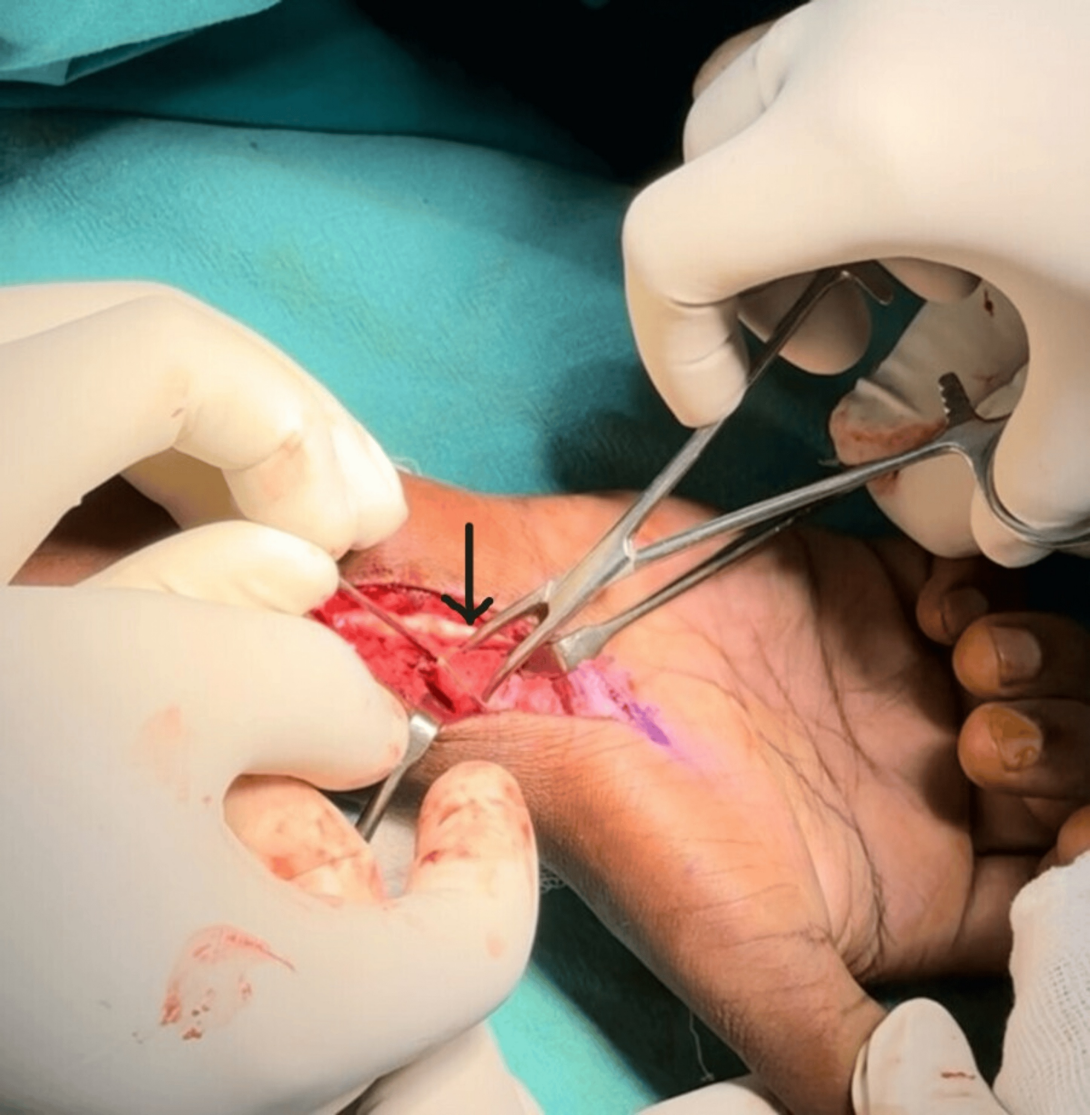
Wedge graft impacted into the nonunion site

**Figure 4:**
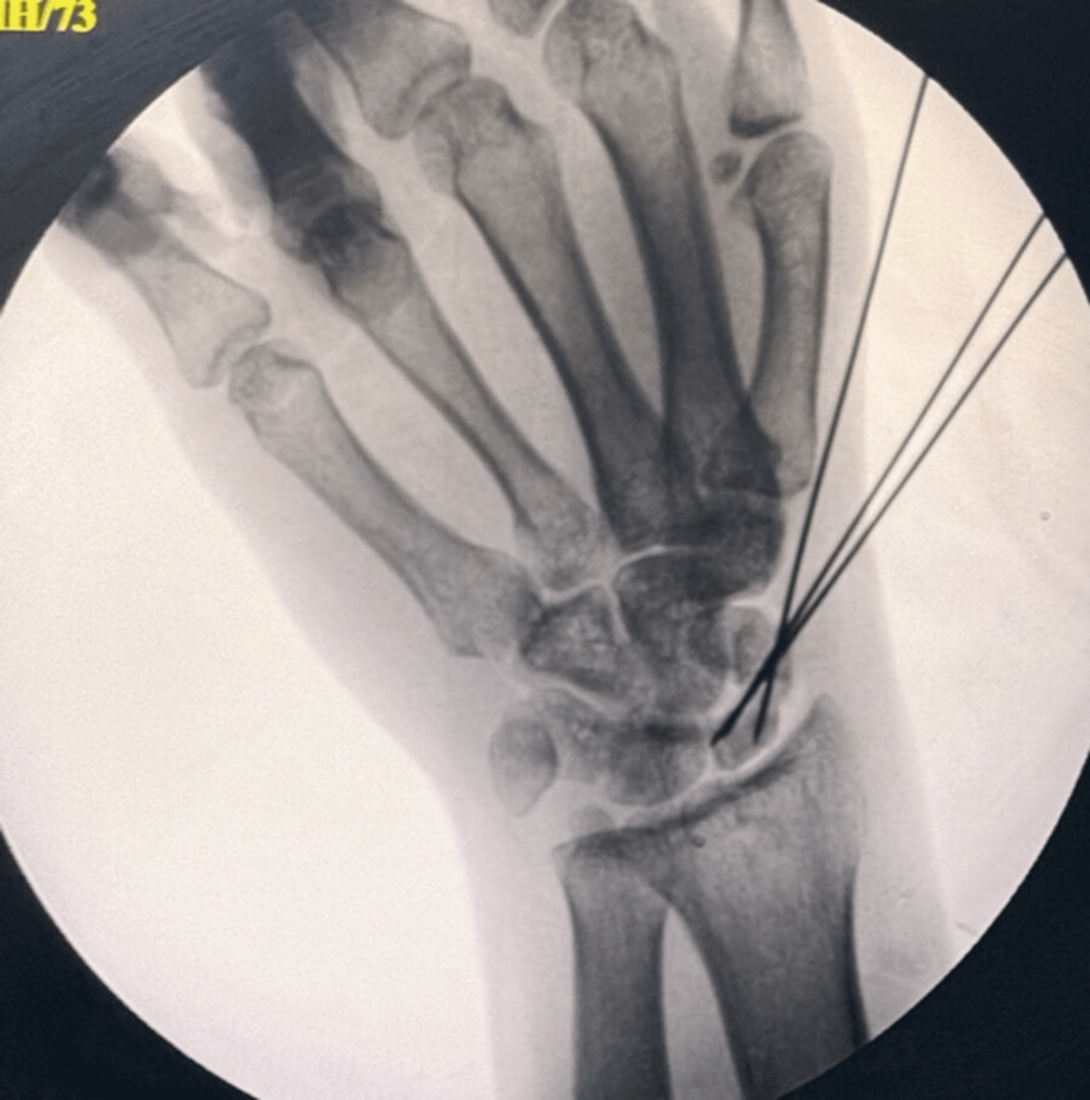
Graft and fragments stabilized with K-wires

The fracture site was identified and confirmed. After that, two K-wires were placed in the proximal and distal poles. These were used to distract the nonunion site (Figure 2). The soft tissues and adhesions were removed using a curette. A trapezoidal wedge graft was harvested from an ipsilateral iliac crest and was shaped using a nibbler. Using artery forceps, the wedge graft was impacted into the gap at the nonunion site, stabilized with a graft punch, and momentarily fastened with K-wires (Figure 3). Subsequently, the fragments and the graft were stabilized using three (1 mm) K-wires (Figure 4).

**Figure 5:**
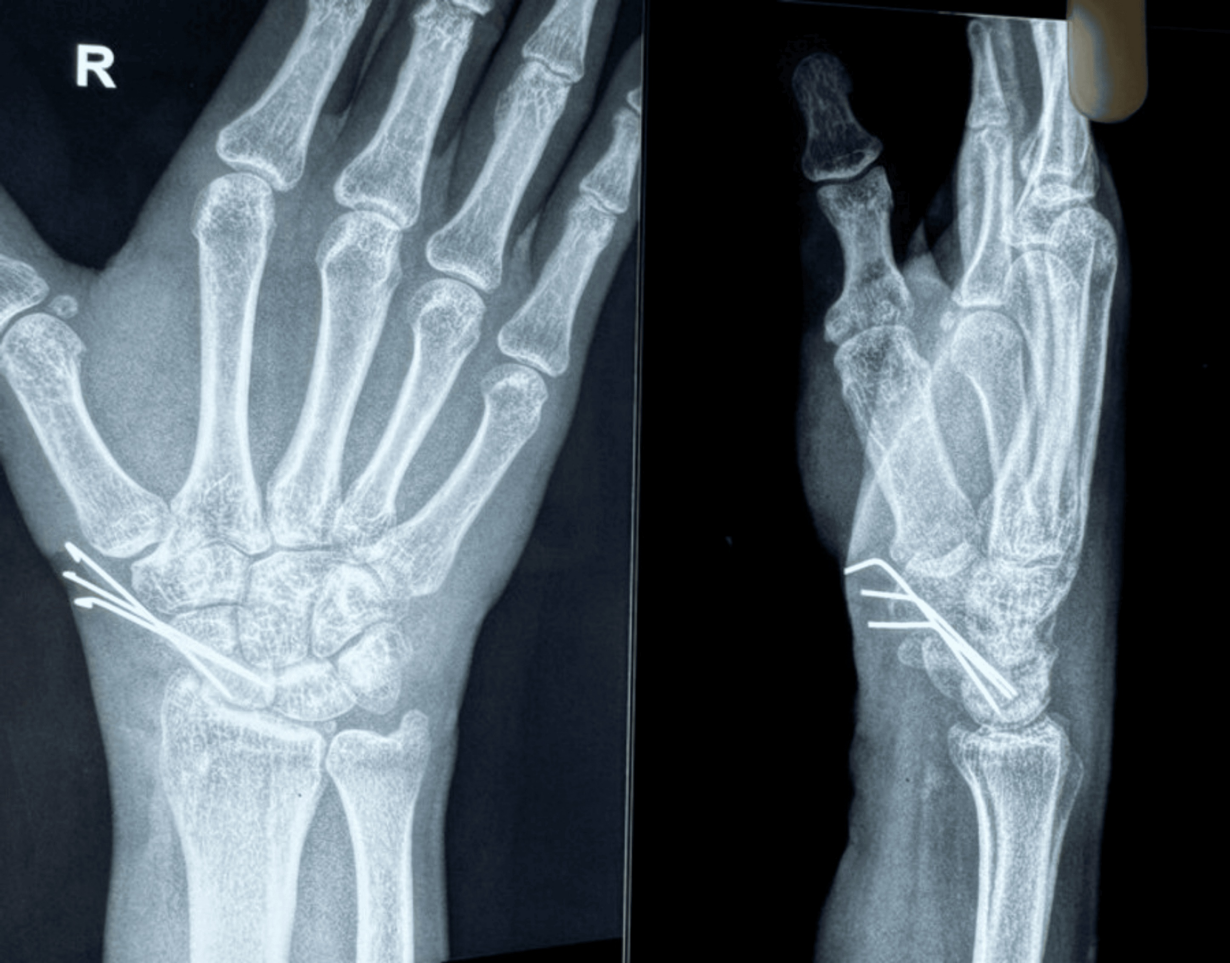
Postoperative X-ray at one month

K-wire placements and joint surface non-penetration were examined in the anteroposterior and lateral views of the wrist using the C-arm image intensifier. A slab was used to support the wrist. Preoperative and postoperative measurements were made for the QuickDASH-9, VAS, and PRWE scores [8,9]. Every surgery was carried out by a single surgeon. All patients were immobilized for four weeks following surgery, and then X-rays were taken (Figure 5). At six, 12, and 24 weeks, clinical results were obtained. Video 1 shows the surgical procedure of scaphoid nonunion.

**Video 1:** Surgical procedure of scaphoid nonunion

## Results

The average time for bone union with K-wires was six months, ranging from four to 18 months. One patient developed an AVN scaphoid and was later treated with wrist arthrodesis. Two patients developed a pin-site infection and were treated with sequential dressing, and two patients developed K-wire loosening. Most patients achieved bony union with K-wires. In the study, the loss of range of motion observed among patients varied from 10° to 30°. On average, patients experienced a reduction of 15° in their range of motion. The preoperative group's mean on the VAS was 71.62 (Table 1) when it was tabulated, and at the 24-week follow-up, it had dropped to 21.52.

**Table 1:** VAS, SD, and SE VAS: visual analog scale, SD: standard deviation, SE: standard error

VAS	Preoperative	Six weeks	12 weeks	24 weeks
Mean	71.62	38.69	29.83	21.52
SD	13.19	5.88	5.45	6.38
SE mean	2.01	0.91	0.84	0.98

The functional outcomes were analyzed using the QuickDASH-9 score. The mean of the patients in the preoperative groups was 87.41. During follow-up appointments, there was a downward trend in the mean QuickDASH-9 score. Six weeks in, the mean score was 73; at 12 weeks, it was 62.79; and at 24 weeks, it was 24.61 (Table 2). The functional outcomes and patient satisfaction were assessed using the PRWE score. The mean of the patients in the preoperative groups was 80.11. The mean score was 60.39 at six weeks, 40.23 at 12 weeks, and 19.98 at 24 weeks. In the preoperative group, the mean PRWE score was 83.45. There was a perpetual decline in PRWE scores in the follow-up visits.

**Table 2:** PRWE PRWE: patient-rated wrist evaluation, SD: standard deviation, SE: standard error

QuickDASH-9 score	Preoperative	6 weeks	12 weeks	24 weeks
Mean	87.41	73	62.79	24.61
SD	4.40	7.85	4.29	5.50
SE mean	0.68	1.21	0.66	0.85
PRWE score	Preoperative	6 weeks	12 weeks	24 weeks
Mean	80.11	60.39	40.23	19.98
SD	2.20	2.06	1.71	2.24
SE mean	0.34	0.32	0.26	0.35

While the quantitative data was analyzed using averages and standard deviations, percentages and frequencies were used to represent categorical variables (Table 3). The means obtained were compared using a paired t-test, where a p-value of less than or equal to 0.05 was deemed statistically significant.

**Table 3:** P-values are statistically significant

QuickDASH-9 score	t-value	p-value
Preoperative/6 weeks	10.42	<0.005
Postoperative/12 weeks	27.90	<0.005
Postoperative/24 weeks	53.43	<0.005

## Discussion

Failure to unite at three months is known as scaphoid delayed union, while failure to unite at six months following scaphoid fracture is known as scaphoid nonunion [10]. The greater number of nonunions and AVN in patients with scaphoid fractures can be attributed to the restricted and retrograde blood supply to the scaphoid proximal pole and the stress vectors acting along the scaphoid fragments during motion, which hinder the healing process [10,11]. To show trends in scaphoid healing, we need to analyze the healing progress of scaphoid fractures over time, typically measured at various intervals such as six weeks, 12 weeks, and 24 weeks. This analysis can include metrics such as the QuickDASH-9 score, pain scores (VAS), and radiographic evidence of healing.

Because scaphoid fractures are mild and manifest with few symptoms, they frequently go undiagnosed at first. Missing these fractures increases the chance of AVN, malunion, or nonunion. Up to 12% of individuals may experience nonunion if the fracture goes undetected [10,11]. The nonunion of the scaphoid is very difficult to treat and can cause osteoarthritis, humpback deformity, and scaphoid nonunion advanced collapse if left untreated. A patient with nonunion typically exhibits a reduction in grip strength, wrist pain, edema, and tenderness at the anatomical snuff box. While cast immobilization is a common treatment for many scaphoid fractures, complex scaphoid fractures usually require bone grafting with internal fixation to avoid wrist complications that could be incapacitating [12].

Fisk proposed that correcting the deformity by inserting a trapezoidal wedge graft would allow a good reduction without distorting the scaphoid length and angulation. Later, Fernandez improved the technique using a wedge graft from the iliac crest followed by internal fixation, achieving similarly successful outcomes [13]. In our study, we find K-wires to be more practical, as using three K-wires traversing the graft from the distal to proximal poles along different axes provides better stability and fixation. We avoided the use of screws to avoid related complications like screw protrusions and guide wire breakage.

There was no statistically significant difference observed in the clinical and radiological outcomes between the Fisk-Fernandez approach, the Matti-Russe technique, and vascularized bone grafting. The quickest method of achieving bone union, however, was vascularized bone grafting. Fisk-Fernandez can provide solid fixation and reduce unintended shearing and rotating pressures during application [14].

Scaphoid length and angulation were addressed by interposition bone grafts. Several divergent pinnings as opposed to screws during the application prevent unintended shearing and rotating stress and offer stable fixation with little dissection.

The functional outcomes were analyzed using the QuickDASH-9 score. The mean of the patients in the preoperative groups was 87.41. During follow-up appointments, there was a downward trend in the mean QuickDASH-9 score. Six weeks in, the mean score was 73; at 12 weeks, it was 62.79; and at 24 weeks, it was 24.61 (Table 1). The functional outcomes and patient satisfaction were assessed using the PRWE score. The mean of the patients in the preoperative groups was 80.11. The mean score was 60.39 at six weeks, 40.23 at 12 weeks, and 19.98 at 24 weeks. In the preoperative group, the mean PRWE score was 83.45. There was a perpetual decline in PRWE scores in the follow-up visits.

Despite the fracture's classification and location, trapezoidal anterior wedge bone grafting combined with internal fixation using K-wires is an effective method. However, the time required for a union is lengthy. It is preferable due to its advantages of cost, ease of insertion, and accessibility [15]. Regardless of the fixation materials utilized, autogenous cancellous grafting can effectively treat scaphoid pseudoarthrosis due to its strong union potential and good vascularity, enabling us to proceed with [16].

In our study, it was found that using K-wires instead of screws gives good results in terms of cost-effectiveness, ease of removal, patient compliance, and fewer complications. This study has several limitations. It is retrospective, and the interventions were all performed by a single trained surgeon, affecting the overall applicability and findings. Furthermore, the study was conducted at a single tertiary center, which might limit its external validity. The data collection relied on history and interview methods, introducing the possibility of recall bias.

## Conclusions

This study reveals that using palmar base anterior wedge grafts to treat carpal scaphoid pseudoarthrosis allows for rectifying scapholunate angle deformities and achieves anatomical restoration in more individuals. Interposition bone grafts were effective in correcting scapholunate angle and scaphoid length. Employing 2-3 K-wires instead of headless screws/lag screws prevents undesirable shearing and rotational forces during the procedure, ensuring solid reduction and promoting bony union. Most of our scaphoid nonunions were oblique varieties, where lag screws failed to achieve perfect compression because of shearing forces.

## Figures and Tables

**Figure 6:**
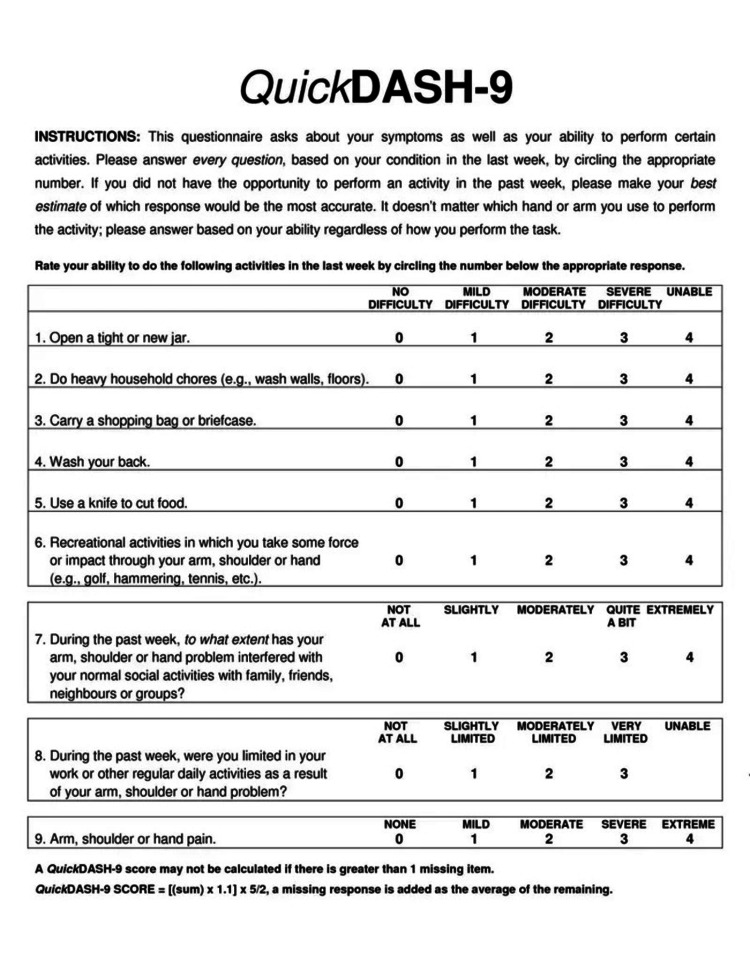
QuickDASH-9 score Image Source: Gabel et al. (2009) [8]

**Figure 7:**
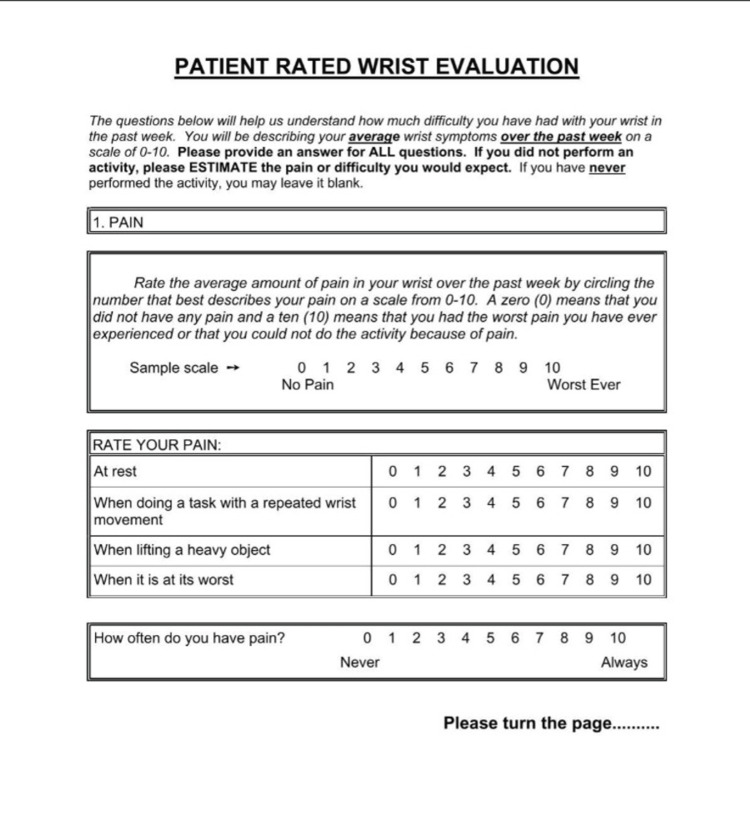
PRWE score page 1 PRWE: patient-rated wrist evaluation Image Credit: Kleinlugtenbelt et al. (2018) [9]

**Figure 8:**
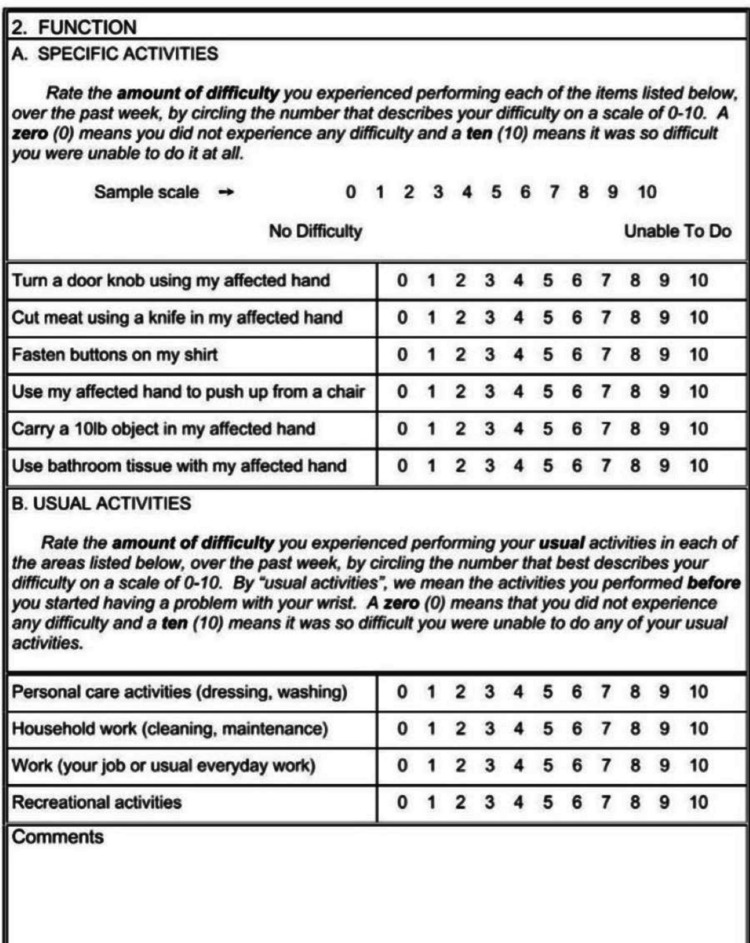
PRWE score page 2 PRWE: patient-rated wrist evaluation Image Credit: Kleinlugtenbelt et al. (2018) [9]
